# Variation in the Content of Three Tandem Repeats of the Human Genome (Ribosomal, Satellite III, and Telomere) in Peripheral Blood Leukocyte DNA of People of Different Ages (5–101 Years)

**DOI:** 10.1155/jare/8847073

**Published:** 2025-09-11

**Authors:** E. S. Ershova, P. E. Umriukhin, R. A. Zinchenko, T. P. Vasilieva, S. E. Kostyuk, N. Yu Shabalin, T. A. Salimova, E. M. Malinovskaya, N. N. Veiko, S. V. Kostyuk

**Affiliations:** ^1^Laboratory of Molecular Biology, Research Centre for Medical Genetics, Moscow, Russia; ^2^Institute of Longevity with a Clinic of Rehabilitation and Preventive Medicine, State Scientific Center of the Russian Federation, Russian Scientific Center of Surgery Named After Academician B. V. Petrovsky, Moscow, Russia; ^3^Federal State Autonomous Educational Institution of Higher Education, I. M. Sechenov First Moscow State Medical University, Ministry of Health of the Russian Federation (Sechenovskiy University), Moscow, Russia; ^4^Department of Lifeway and Public Health Protection Investigation, N. A. Semashko National Research Institute of Public Health, Moscow, Russia; ^5^Faculty of Science, RUDN University, Moscow, Russia

**Keywords:** aging, centenarians, CNVs, human ribosomal genes, satellite III (1q12), telomere repeat

## Abstract

The variation of ribosomal (parameter **R**), satellite III (1q12) (parameter **S**), and telomere (parameter **T**) tandem repeats content of the human genome was studied in DNA samples isolated from blood leukocytes of 535 people whose age varied from 5 to 101 years. For analysis we used the method of nonradioactive quantitative hybridization. The group of centenarians (90–101 years old, *N* = 106) differs from other age groups by a significantly narrower distribution of the ribosomal repeat content in DNA, a much higher content of satellite III, and a lower content of telomere repeat. A negative correlation was found between the **S** and **T** parameters (*p* < 10^−4^). The findings of this study suggest that the calculated parameters **S/T** and **S/(R∗T)** exhibit a marked increase with age, culminating in maximal values within the cohort of centenarians. These results imply that the parameters **R**, **S/T**, and **S/(R∗T)** may hold the potential to serve as reliable predictors of life expectancy for individuals in advanced age.

## 1. Introduction

About 70% of all human genome sequences are occupied by repetitive DNA sequences (repeats) [1]. Tandem repeats represent a significant part of the total number of genome repeats [2]. Tandem repeat units are arranged in a head–tail pattern and can be repeated many times, forming extended clusters in certain DNA regions. A distinctive feature of tandem repeats is their pronounced qualitative and quantitative polymorphism. Tandem repeats perform a variety of cellular functions and are associated with many human diseases [3–9].

Due to the lack of methods acceptable for tandem repeats analysis, the variation of tandem repeats copies number in the composition of long clusters localized on different chromosomes has not been sufficiently studied to date. As PCR and various sequencing methods do not allow us to reliably determine the number of copies of the tandem repeat in DNA, these DNA sites are classified as areas with a poor sequencing level (“gray zones” of the genome) [10].

Only in recent years, due to the development of the long DNA fragment sequencing method not using PCR (Oxford nanopore), single data began to appear in the literature reflecting the qualitative and quantitative composition of extended clusters of tandem repeats of the human genome [11]. Previously, we proposed a quantitative method (nonradioactive quantitative hybridization [NQH]) allowing us to determine the total content of long fragments of highly repetitive tandem repeats in human DNA [12]. The method is based on the hybridization of nonradioactively labeled DNA probes with denatured DNA immobilized on a nitrocellulose membrane. The data obtained using a new method for long DNA fragments sequencing completely confirmed the results obtained earlier by NQH, reflecting the copy number variation (CNV) of the ribosomal repeat in the genome [11]. Using NQH, we consequently studied the ribosomal repeat content [12, 13], satellite fragment III (1q12), and telomere repeat in DNA isolated from leukocytes of the blood of people under the age of 90 years and from cultured human cells [14]. These repeats perform various cellular functions, but they share a common property: Variation of these repeats' content in the genome significantly affects the architecture and stability of the cell nucleus chromatin. At the same time, cell aging is primarily associated with changes in the genome expression profile, caused by changes in the structural organization and instability of the nuclear chromatin [15–19].

### 1.1. Ribosomal Repeat

**Figure 1:**
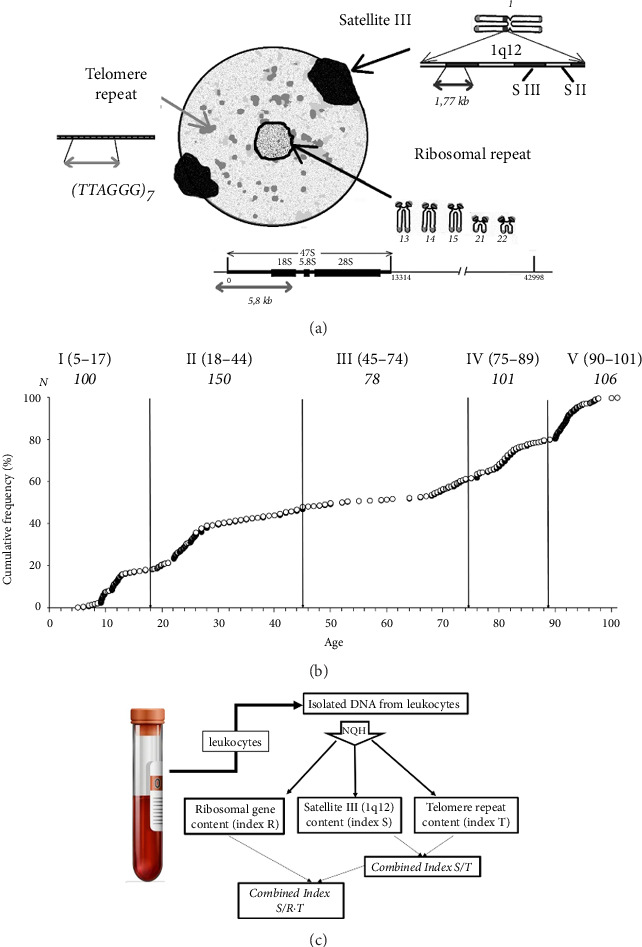
(a) Scheme of analyzed tandem repeats of the human genome. **Ribosomal repeat** (p-regions of acrocentric chromosomes): The transcribed region (47S) is indicated, which contains genes for 18S, 5.8S, and 28S rRNAs and transcribed spacers. The DNA probe for rDNA contained a fragment of the transcribed region (5836 bp, positions from −515 to 5321 bp). **Satellite III (1q12)**: The probe to satellite III is a fragment of region 1q12 with a length of 1.77 Kb [20]. **Telomere repeat:** located at the ends of all chromosomes. The arrangement of repeats in the interphase nucleus is provided according to the previous publications [21, 22]. (b) A histogram reflects the division of the sample into the groups according to the DNA donors' age. (c) Experimental design. The parameters determined in the experiment and the calculated parameters are indicated.

The human genome contains hundreds of rDNA copies arranged in tandem repeats on five acrocentric chromosome pairs (14, 15, 13, 21, and 22). The repeat includes a transcribed region and a nontranscribed intergenic spacer (Figure 1(a)). The transcribed repeat region encodes 47S pre-RNA, which is further processed to form 18*S* rRNA (small ribosome subunit component) together with 5.8*S* and 28*S* rRNAs (large ribosome subunit components). In the interphase nucleus, rDNA repeats form the nucleolus structure, where rDNA transcription and the initial ribosome biogenesis stages take place. Four rDNA subfractions are determined in the nucleolus, which differ by transcription activity at a given time, methylation level, and the presence of nucleosomal organization: (1) active and (2) potentially active unmethylated copies (30%–50% of the total number of copies); (3) methylated inactive copies in the promoter region; and (4) hypermethylated rDNA copies localized outside the nucleolus. Hypermethylated copies are present only in 5%–10% of human DNA samples [23–34]. The number of active rDNA copies in the nucleolus is proportional to the total number of rDNA copies, except in cases when hypermethylated copies are present [27].

Ribosomal repeats in the nucleolus represent a kind of center regulating the entire chromatin stability of the nucleus and affecting the lifespan of the cell [35–37]. It has been shown that ribosomal repeats have thousands of contacts with other chromatin sites in the interphase nucleus and can regulate activity of many genes [38–43]. Earlier, we analyzed the change in the number of rDNA copies in the genomes of people aged 16–90 years and found an interesting regularity [13]: After 72 years (average life expectancy in Russia), there is a significant narrowing of the distribution of DNA samples by the number of rDNA copies. The question arises: How pronounced is this range narrowing in the group of centenarians (people over 90 years old), and can this index be used as a predictive longevity marker?

### 1.2. Satellite III (1q12)

Region 1q12 contains the largest centromeric heterochromatin fragment in the human genome, which consists of long tandem repeats of satellites II and III [44]. Demethylation and decondensation of satellite III is an early sign of cellular aging [45–47]. The satellite III (1q12) chromatin region is very susceptible to DNA double helix damage, while the efficiency of this region's repair is significantly reduced [48]. It results in genomic instability of this site in cancer and a number of other diseases [49–54]. Cellular stress and aging induce satellite III (1q12) transcription [21, 46].

During interphase, the 1q12 region detected by the satellite III probe [20] is localized on the periphery of the nucleus [21]. During the cell cycle, this fragment approaches and contacts the rDNA in the nucleolus [55]. Similar chromatin changes are observed in cells during adaptive response development to oxidative stress [21]. The absence of 1q12 site transposition toward the nucleolus is associated with a block of the adaptive response and an increased level of cellular death. These events correlate with the satellite III content increase in the discussed DNA fragment [55]. Earlier, for the first time, we found an increase in the satellite III content during the replicative aging of skin cells and analyzed the satellite III content dynamic in people under 90 years of age [14]. In the elderly group (75–90 years old), the biphasic distribution of DNA samples according to satellite III content was found. A tendency was observed for higher repeat content in the DNA of people over 85 years of age. It can be assumed that the genome of centenarians contains the largest amounts of satellite III. In such a case, the high satellite III content in the elderly may serve as a prognostic indicator of a longer life.

### 1.3. Telomere Repeat

DNA sequences at the ends of human chromosomes are represented by tandem repeats (TTAGGG)_n_, which create a buffer that determines the number of divisions that a cell can undergo [56]. Single-strand break repair mechanisms are poorly developed in telomeric DNA, unlike genomic DNA [57]. The two main signs of physiological aging in humans include loss of genome integrity and a telomere length decrease, leading to disruption of the architecture of nuclei and mistakes during replication [22]. In recent years, an increasing amount of data has been acquired on the structural and functional relationship of telomere and ribosomal repeats in the cell nucleus [58].

A decrease in telomere length is associated with the decondensation and transcription of heterochromatin sites (including centromeric heterochromatin that involves satellite III) and with the development of a number of diseases caused by the human body aging [59–73]. Previously, we found that the telomere repeat content in DNA negatively correlates with the satellite III content during replicative aging and in various human brain structures [14, 74].

Therefore, all three tandem repeats interact in the cell, affecting the chromatin structure and hence the gene expression profile in the nucleus. The role of these repeats in the functioning of the cell largely depends on the size of the repeat clusters, i.e., on their copy number in the nucleus. It is of great interest to compare the content of a widely accepted aging marker (telomere repeat) with the content of two other potential markers of aging (ribosomal and satellite III) in the DNA of centenarians.

The purpose of the present study is to analyze the variation in the number of three tandem repeats of the human genome (ribosomal, satellite III (1q12), and telomere) in DNA isolated from the blood leukocytes of people of different ages without obvious genetic pathologies. Special attention was paid to the group of centenarians, for whom such studies had not been conducted before.

## 2. Materials and Methods

### 2.1. Inclusion and Exclusion Criteria for the Participants

DNA samples from 429 residents of Moscow and the Moscow region aged 5–90 years (56% men) participated in the study. These samples were isolated from blood leukocytes earlier and stored at −80°C in the collection of DNA samples from the Laboratory of Molecular Biology of the Research Centre for Medical Genetics. 106 DNA samples were isolated from peripheral blood leukocytes of centenarians aged 90–101 years (35% men) during this study. All the centenarians lived in the Moscow House of War Veterans. Clinical and demographic characteristics of experimental groups are presented in Table 1.

Inclusion and exclusion criteria for the participants. The participants must not have obvious genetic disorders or any relatives with genetic pathology. They also should not have chronic diseases that significantly reduce life expectancy (autoimmune pathologies, mental disorders, neurodegenerative diseases, dementia of various origins, etc.).

Informed consents were obtained from participants over the age of 16. The parents of the participants aged 5–16 years gave informed consent for their child's DNA analysis. The study design was approved by the ethics committee of the Federal State Budgetary Scientific Institution “Research Centre for Medical Genetics” (protocol # 6/4 dated 15.11.2016).

### 2.2. Isolation of DNA From Leukocytes

A volume of 5 mL of blood was taken from the peripheral vein into a tube with heparin (0.1 mL/5 mL of blood). Leukocytes were isolated from 5 mL of blood according to the standard protocol [75]. The standard method described in detail earlier was used to isolate DNA [12]. The method includes cell lysis (2% sodium laurylsarcosylate, 0.04 M EDTA), RNAse processing (150 μg/mL, 45 min, 37°C; Sigma, USA), and proteinase K treatment (200 μg/mL, 24 h, 37°C; Promega, USA). The proteins were extracted with phenol, phenol-chloroform (1:1), and a mixture of chloroform-isoamyl alcohol (24:1). DNA from an aqueous solution was precipitated (with the addition of 0.3 M sodium acetate) with 2 volumes of ethanol. The DNA precipitate was washed with 70% ethanol and dissolved in water. The DNA concentration was determined by two methods—by measuring the absorption spectra and then, more precisely, using the PicoGreen dye fluorescing in combination with DNA (molecular probes, Invitrogen, California, USA).

### 2.3. The NQH

The method was described in detail earlier [12, 14, 76] and was used unchanged. The DNA probe for rDNA contained an *Eco*RI fragment of the transcribed region (5836 bp, positions from −515 to 5321 bp; GenBank No. U13369) cloned into the plasmid pBR322. The probe to satellite III is a fragment of region 1q12 cloned into an *Eco*RI plasmid with a length of 1.77 Kb (known as pUC1.77 [20]). Dr. H. Cook (MRC, Edinburgh, UK) kindly provided this probe. DNA probes were labeled with biotin by nick translation using biotin-11-dUTP. As a telomere repeat probe, we used biotinated 5-terminal oligonucleotide (TTAGGG)_7_ that was synthesized by Syntol (Moscow).

### 2.4. Statistical Data Processing

Four parallel samples of each DNA specimen were applied to the filter in every experiment. The experiment was repeated 2–3 times. To determine the number of repeats based on the results of hybridization we used the program “Image 6.0” (Research Centre for Medical Genetic).  Table 2 provides descriptive statistics for three parameters reflecting the content of repeats in DNA. The distributions of the studied parameters were analyzed using the cumulative curve method. Cumulative distribution describes the probability that a random variable is less than or equal to a certain value. It is a function that accumulates the probabilities from the smallest values of a random variable up to a given value.

Table 3 compares the age groups using the methods of nonparametric Mann–Whitney statistics (comparison of groups by repeats content), Kolmogorov–Smirnov (comparison of sample distributions by repeats content), and receiver operating characteristic (ROC) analysis. MedCalc (https://www.medcalc.org/manual/roc-curves.php) produced a complete sensitivity/specificity report (ROC curve analysis). Each point of such a curve represents a sensitivity/specificity pair corresponding to a particular decision threshold. The area under the ROC curve (AUC) shows the parameter difference between two compared groups.

Spearman's statistics were used to analyze correlations between values. The data were analyzed using the StatPlus2007 Professional software package (https://www.analystsoft.com). ROC analysis was performed using MedCalc software (https://www.medcalc.org/manual/roc-curves.php).

## 3. Results

DNA samples were isolated from peripheral blood leukocytes of 535 people aged 5–101 years. The study participants and their relatives had no obvious genetic diseases. The age distribution of the study participants is shown in the cumulative histogram on Figure 1(b). The entire sample was divided into 5 age groups: I (5–17 years, *N* = 100), II (18–44 years, *N* = 150), III (45–74 years, *N* = 78), IV (75–89 years, *N* = 101) and V (90–101 years, *N* = 106). The content of three repeats in the isolated DNA samples was determined by NQH which was specially developed for the tandem repeats analysis in the human genome [12, 14, 76]. DNA probes indicated in Figure 1(a) were used for hybridization to identify fragments of ribosomal, telomeric and satellite repeats. The raw data for each individual are presented as a function of age in the Supporting.

### 3.1. Ribosomal Repeat

**Figure 2:**
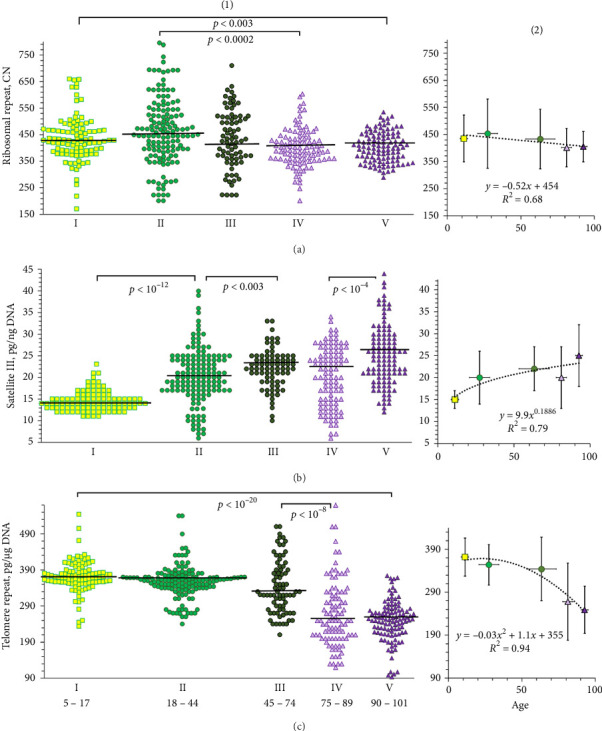
Experimental data reflecting changes in the content of three tandem repeats in the DNA of people from different age groups. (a(1), b(1), c(1)): The content of ribosomal, satellite, and telomere repeats in different age groups. The horizontal lines represent median values. The groups are compared using the Mann–Whitney method. (a(2), b(2), c(2)): Dependence of the average content of ribosomal, satellite, and telomere repeats on the average age in the group. The figure shows the standard deviation and trend lines most accurately approximating the corresponding dependencies.

**Figure 3:**
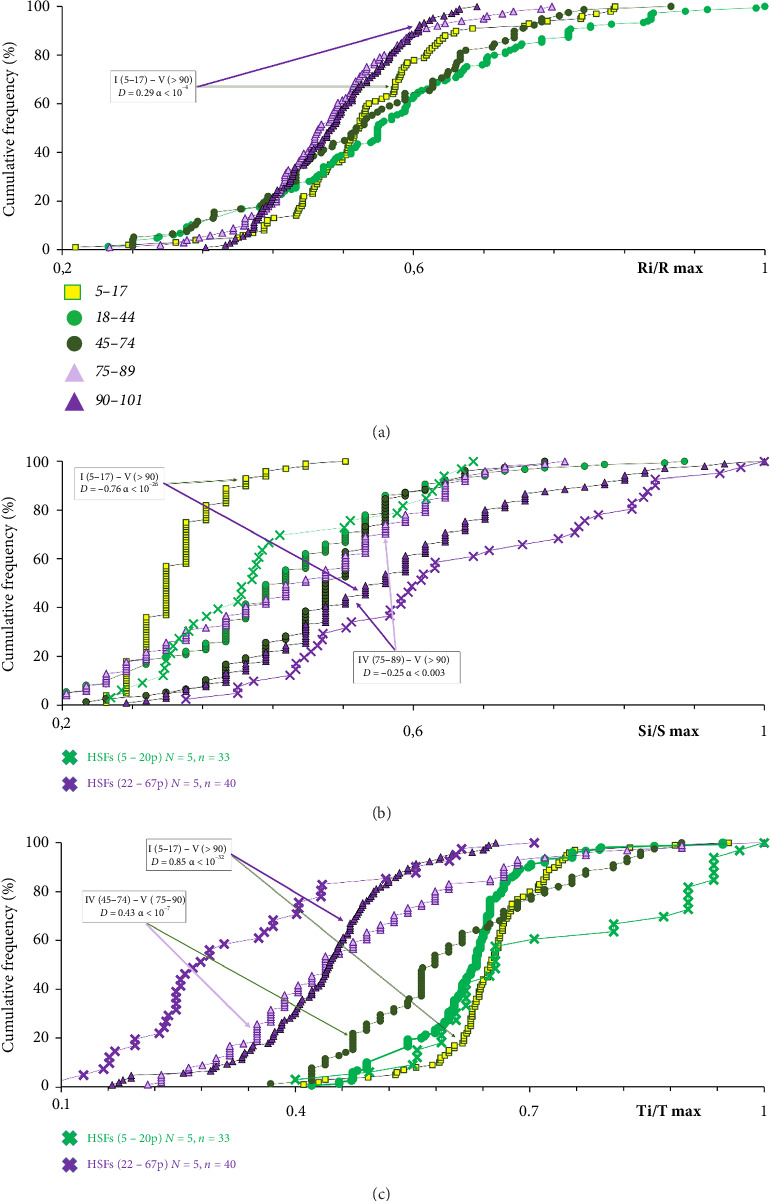
Cumulative distributions of DNA samples from different age groups according to the content of ribosomal repeat (a), satellite III repeat (b), and telomere repeat (c). Parameter values normalized to the maximum values of these parameters in the sample are placed on the *X*-axis. Graphs (b and c) include previously obtained data in experiments with replicative aging of five lines of cultured skin fibroblasts from five donors of different ages. The values for cells of early passages (5–20 passages) and late passages (22–67 passages) are compared [14].

The rDNA content was expressed as the number of the repeat of per diploid genome (parameter **R**).  Figure 2(a)(1) shows experimental data reflecting rDNA content in DNA samples from different age groups.  Figure 2(a)(2) shows the average **R** parameter values and the standard deviations for five age groups.  Table 2 provides descriptive statistics for **R** index.  Figure 3(a) shows the cumulative distributions of DNA samples from five groups by the number of rDNA copies. Comparison of I–V groups by the R, S and T values is presented in Table 3.

The number of rDNA copies in the sample ranged from 171 copies to 796 copies per diploid genome. Age groups I, II, and III (5–74 years old) do not differ in terms of rDNA content. Groups IV and V of older age (75–101 years) also do not differ from each other in terms of rDNA content and by the distribution of samples by R index. The number of rDNA copies in the genome negatively correlated with age (Spearman coefficient Rs = −0.19; *p* < 10^−5^; *N* = 535, Supporting). The dependence of R on age for the entire sample is approximated by a linear equation (*y* = −0.6*x* + 459; *R*^2^ = 0.04; *N* = 535, Supporting).

The older age groups IV and V (75–101 years) differ from the younger age groups I-III (5–74 years) by reduced (by 7%) average **R** values, a narrow **R** range, and a lower coefficient of variation (Table 2). These changes were most expressed in the group of centenarians (90–101 years). The number of rDNA copies in the group of centenarians varies in a narrow range from 289 to 535 copies. The range of **R** values for the group of centenarians is reduced 2–2.4-fold compared with groups I–III and 1.6-fold compared with the elderly group IV. In the younger age groups (5–74 years, *N* = 328), 9% of DNA samples contain less than 289 rDNA copies, and 18% of samples contain more than 535 rDNA copies. We found no significant differences of parameter **R** between DNA samples isolated from the blood of men and women.

Thus, the natural process of human aging is accompanied by a subtle reduction in the average content of rDNA content in leukocyte DNA, as well as a marked decrease in the range of variation of the R index variation range in the cohort of centenarians.

### 3.2. Satellite III (1q12)

The content of satellite III is presented in picograms of repeat content (1.77 kb long) in 1 ng of DNA (parameter **S**) [14]. A quantity of 1 pg of repeat/ng of DNA corresponds to approximately 1600 copies of repeat per diploid genome.  Figure 2(b)(1) shows experimental data reflecting the satellite III content in DNA samples of different age groups.  Figure 2(b)(2) shows the average values of the parameter **S** and the standard deviation for five age groups.  Table 2 provides descriptive statistics for **S**.  Figure 3(b) shows the distributions of DNA samples from five groups according to the values of parameter **S**. In Table 3, the age groups are compared with each other.

The average content of satellite III in DNA ranges from 15 ± 2 (group I) to 25 ± 7 (group V) pg repeat/ng DNA. We found no significant differences in the value of parameter **S** between DNA samples isolated from the blood of men and women. The minimal repeat number is found in the younger age group. For this group, a significant decrease of the range and coefficient of variation for S is observed (Table 2). The **S** parameter correlates positively with age (Rs = +0.47; *p* < 10^−15^; *N* = 535, Supporting). However, analysis of the correlation of the parameter with age in individual groups showed that only in the group of children is there a positive correlation of S with age. During 1 year, the satellite III content in children's DNA increases by an average of 0.52 pg/ng of DNA, which corresponds to the average content increasing by ≈ 800 copies or 1.4·10^6^ nucleotide pairs.

The DNA of adults contains increased amounts of satellite III. Against the background of higher parameter **S** average values, there is a significant increase in the range and coefficients of variation. In 18–101-year-old groups (*N* = 435), 9% of DNA samples contain less than 11 pg repeat/ng of DNA, and 39% of DNA samples contain more than 23 pg repeat/ng of DNA. The group of centenarians differs from other groups by the highest **S** parameter values and the absence of DNA samples with very low parameter values (less than 12 pg/ng of repeat DNA). In group IV (75–89 years old), the number of such samples is 17%.

For comparison, Figure 3(b) shows previously obtained data reflecting changes in parameter **S** during replicative aging of cultured skin fibroblasts from five adult donors [14]. DNA samples isolated from cells of early passages (5–20 passages) and late passages (from 22 to 45–67 passages) were compared. The previously described age-related changes in the S parameter during replicative aging of cultured fibroblasts are identical to the changes in S that we observed when comparing groups of young and old people.

Therefore, aging is accompanied by satellite III (1q12) content increase in the DNA of cultured as well as human body cells. The maximal changes in blood leukocytes occur during the transition from childhood (adolescence) to adulthood (over 18 years old). The maximum satellite III amounts are contained in centenarians' DNA samples.

### 3.3. Telomere Repeat

The content of the telomere repeat is presented in picograms of repeat per 1 μg of DNA (parameter **T**) [77]. 1 pg of repeat/μg of DNA corresponds to an average telomere length of 17 bp.  Figure 2(c) shows experimental data reflecting the telomere repeat content in different age groups.  Figure 2(c)(2) shows the average **T** parameter values and the standard deviation for five age groups.  Table 2 provides descriptive statistics for the parameter **T**.  Figure 3(c) shows **T** value distributions in the five groups of DNA samples. In Table 3, the age groups are compared with each other.

The average telomere repeat content in the groups ranged from 249 ± 55 pg/μg of DNA (group V) to 372 ± 44 pg/μg of DNA (group I). The parameter **T** is negatively correlated with age (Rs = −0.62, *p* < 10^−20^, *N* = 535, Supporting). The dependence of **T** on age is approximated by a linear equation (*y* = −1.4*x* + 393; *R*^2^ = 0.32). During 1 year, the telomere repeat content decreases by an average of 1.4 pg/μg of DNA, which corresponds to the average telomere length decrease of 23.6 nucleotide pairs. This value correlates well with the literature data. The review authors provide data on average telomere length decrease by 23 bp per year [78].

The minimum coefficients of variation (0.12–0.13) of the parameter **T** were observed for younger age groups (5–44 years). In the older groups (45–101 years old, *N* = 284), 29% of DNA samples contain less than 234 pg of repeats/μg of DNA, and only 1 sample (0.3%, 78-year-old woman) contains more than 544 pg of repeats/μg of DNA. We found no significant differences in the **T** parameter values of age groups between men and women.

For comparison, Figure 3(c) illustrates previously obtained data reflecting **T** parameter changes during the replicative aging of cultured skin fibroblasts from five adult donors [14]. DNA samples isolated from early passages (5–20 passages) and late passage cells (22-45-67 passages) were compared. The age-related **T** parameter changes observed in human leukocytes are identical to the previously described changes that occur during the replicative aging of cells.

Therefore, in the studied sample, we confirmed numerous findings from other authors regarding a decrease in the telomere repeat content (average telomere length) during natural and replicative aging of human cells.

### 3.4. Combined Analysis of the Variation of Three Repeats Content in the DNA of Leukocytes

**Figure 4:**
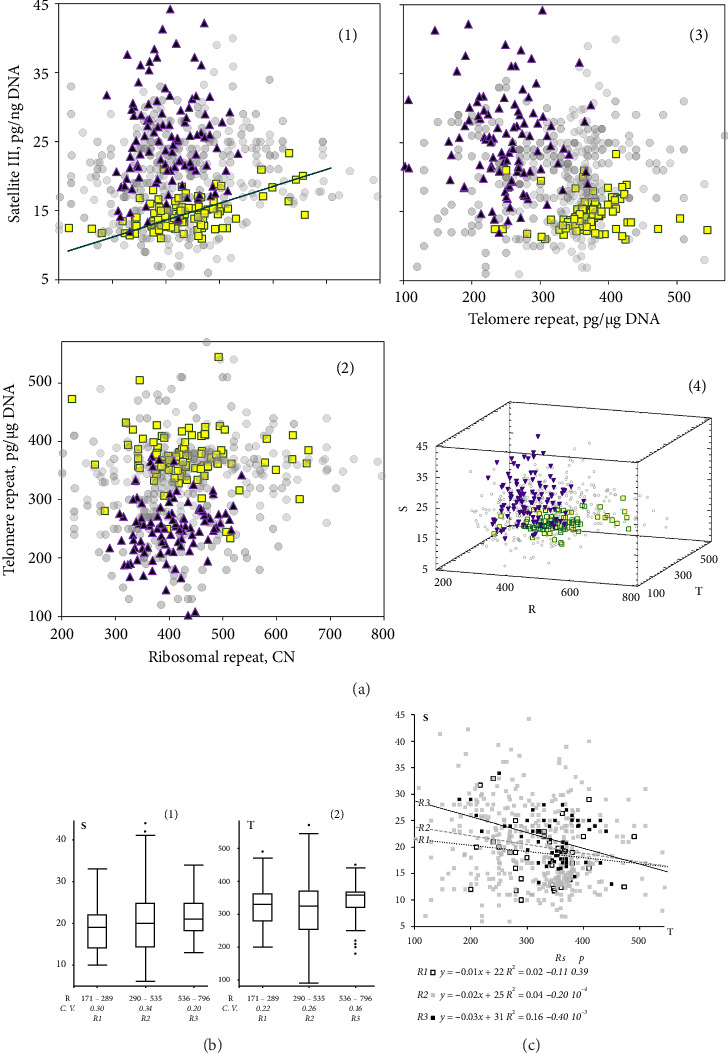
(a(1–4)). The dependence of the content of three tandem repeats in human DNA samples. The younger group I (white circles) and the older group V (black triangles) are marked separately. Groups II–IV are indicated by gray circles. (b) The values of the **S** (1) and **T** (2) parameters in the subgroups formed by the number of rDNA copies in the genome. Descriptive statistics are provided by box plot diagrams, which show the median, lower and upper quartiles, minimum and maximum parameter values in the subgroup, and outliers. (c) Dependence of the **S** parameter on **T** in the R1–R3 subgroups formed by the number of rDNA copies in the genome. The linear regression equations are shown in the frame.

Figure 4(a) (1–3) depicts the dependencies of two repeats of content in DNA samples. Figure 4(a) (4) illustrates the dependency of three repeats of content in DNA concurrently. The graphs highlight the younger group (yellow squares) and the centenarians' group (purple triangles).

#### 3.4.1. Ribosomal Repeat—Satellite III

For the entire sample, there is no significant correlation between the parameters **S** and **R** (*p* > 0.08). For the younger group, I satellite III content in DNA positively correlates with the number of rDNA copies (Rs = +0.38; *p* < 10^−4^, *N* = 100). The more the rDNA copy number in the genome, the greater the satellite III content in DNA isolated from leukocytes. For age group IV (75–89 years old), there is also a positive correlation between the parameters **S** and **R** (Rs = +0.34; *p* < 10^−3^, *N* = 101).

We divided the entire sample into three subgroups: R1 (< 290 copies, *N* = 34), R2 (“centenarians range,” 289–535 copies, *N* = 435), and R3 (> 535 copies, *N* = 66). In the subgroup of DNA samples with a high rDNA content (R3), the coefficient of variation of parameter **S** is lower than in the rest of the sample [Figure 4(b)(1)].

#### 3.4.2. Ribosomal Repeat—Telomere Repeat

For the entire sample, a weak positive correlation was found between the parameters **T** and **R** (Rs = +0.17; *p* < 10^−3^, *N* = 535). As in the case of parameter **S**, the R3 subgroup with a high rDNA content does not contain DNA samples with high and low telomere repeat content. The coefficient of variation of the parameter **T** in the subgroup of samples with a high rDNA content is less than in the rest of the sample (Figure 4(b)(2)).

#### 3.4.3. Satellite III—Telomere Repeat

The content of satellite III negatively correlates with the telomere repeat content (Rs = −0.20; *p* < 10^−4^, *N* = 535). The dependence of the parameters **S** and **T** is approximated by a linear equation (*y* = −0.016 *x* + 25; *R*^2^ = 0.04; *N* = 535).

Figure 4(c) shows the relationship between the **S** and **T** parameters in the R1–R3 subgroups, which were separated by the number of rDNA copies. As the rDNA content increases, the coefficient Rs, reflecting the negative correlation between the content of telomere and satellite repeats, increases.

#### 3.4.4. The Ratio of **S/T** and **S/(R∗T)**

The increase in satellite III content against the background of a decrease in telomeric repeat and rDNA content that we found in older age groups allows us to assume that the calculated parameters **S/T** or **S/(R∗T)** will change to a greater extent with aging than the initial parameters **S, R,** and **T**.

**Figure 5:**
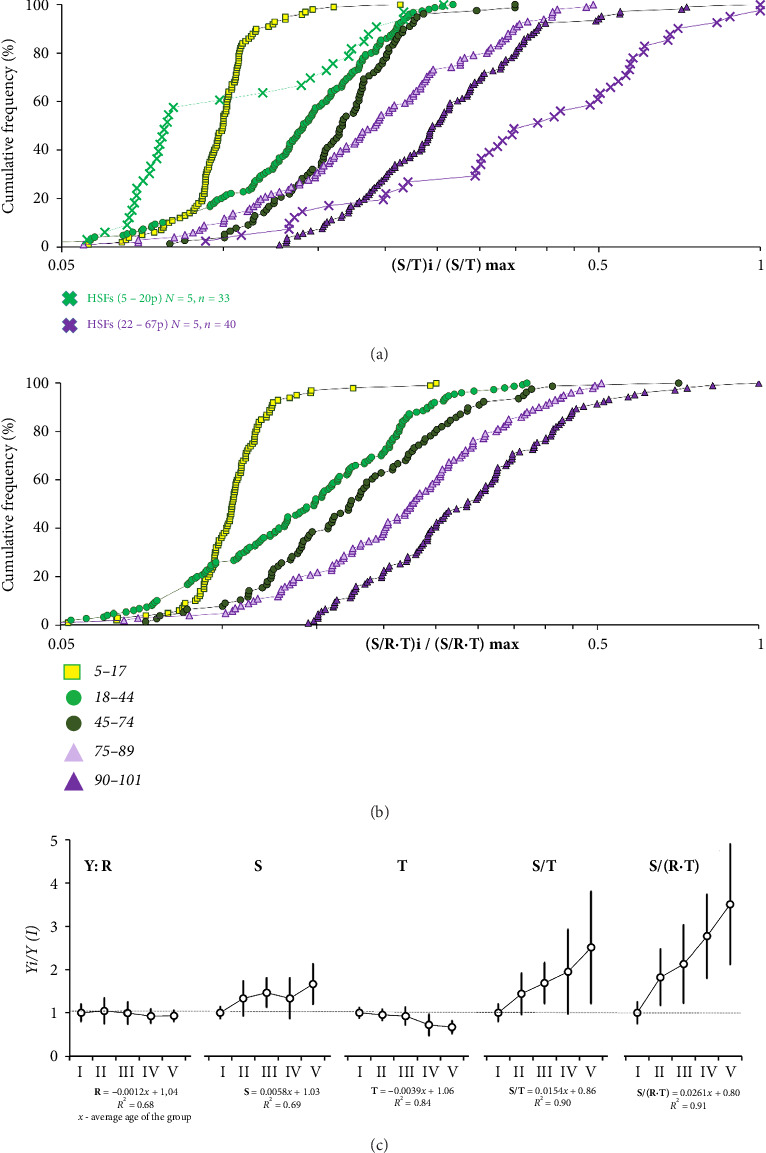
Cumulative distributions of DNA samples from different age groups according to the values of the calculated parameter **S**/**T** (a) and parameter **S**/(**R**∗**T**) (b). The values of the calculated parameters normalized to the maximum values of these parameters in the sample are placed on the *X*-axis. For comparison, graph A also shows previous data of replicative aging of five lines of cultured skin fibroblasts from five donors of different ages. The values for DNA of early passage cells (5–20 passages) and late passages (22–67 passages) are compared [14]. (c) Relative changes of **R**, **S**, **T**, **S**/**T,** and **S**/(**R**∗**T**) indices in age groups. All values are normalized to parameter values in the younger group I (5–17 years old). The standard deviations are shown. Linear regression equations are provided below the graph, reflecting the dependence of the parameter on the average age of the group.

Figure 5(a) shows the **S**/**T** distribution representing the ratio of satellite III and telomere repeat content in the DNA samples. For comparison, the graph also presents previously obtained data reflecting **S/T** ratio changes during replicative aging of cultured skin fibroblasts from five adult donors [14]. As it is shown, the age-related changes of the **S**/**T** index calculated for human leukocytes are identical to the previously described index dynamic detected during replicative aging of skin cells.

The **S**/**T** ratio represents the differences between age groups much more remarkably than **S** or **T** parameter separately (Table 3 and Figure 5(c)). The coefficient of correlation of this parameter with age exceeds the coefficients for the parameters **S** and **T**. Even greater dependence on age is demonstrated by the calculated index **S/(R∗T)**, which takes into account, along with the parameters **S** and **T**, rDNA content. This parameter practically does not overlap in centenarians and the younger groups (Figures 5(b) and 5(c)).

## 4. Discussion

### 4.1. Variation of Three Tandem Repeats Contents in the Human Genome During Aging

In the present study, we analyzed changes in the three tandem repeats content of the genome in the DNA of leukocytes of people of different ages, focusing on previously uninvestigated senior persons, including 106 centenarians aged 90–101 years. Ribosomal, telomere, and satellite repeats differ significantly by the dynamics of their copy number changes in the different age groups (Figures 2 and 3). The main differences between the centenarian group V and the rest of the sample are summarized below. The centenarian's group is characterized by:

1. The maximal narrowing of the rDNA CN (parameter **R)** variation range in comparison with other age groups. The **R** range begins to narrow after 72–74 years (the age of average life expectancy in Russia), and it reaches a minimum among centenarians. Among centenarians, there are no people with high (more than 535 copies) or low (less than 290 copies) rDNA content. In the rest of the sample, **R** values of 27% of DNA specimens are outside the “centenarians' range;”
2. Maximum increase in the average parameter **S** values (by 80% compared to the younger group). The minimum content of satellite III and the narrowest range of variation were observed in the younger age group (Figures 2 and 3). Growing up is accompanied by a sharp satellite III content increase in the leukocytes' DNA. The variation range of parameter **S** increases significantly in groups II–V compared to the younger group. Adult DNA contains predominantly a lot of satellite, but 5% of DNA samples in groups II–IV contain low amounts (less than 10 pg/ng of DNA), even compared to group I. However, DNA samples with such a low **S** parameter value are not found in the group of centenarians;
3. A decrease in the average values of the parameter **T** compared to groups I–III. A significant decrease in the telomere repeat content was observed after 75 years. Group V of centenarians differs from group IV by the absence of DNA samples with a telomere repeat content of more than 374 pg/μg of DNA, which corresponds to an average telomere length of 6.4 kb. These data correlate well with data from other authors [60].

To determine whether the content of any repeat in the human genome changes throughout life, it is necessary to examine DNA samples of the same people at different periods. Unfortunately, we did not have such an opportunity. The approach we exploited, involving a comparative analysis of the repeat content of different people in different age groups, does not allow us to make an unambiguous choice between two hypotheses. The repeated content differences in younger and older groups may be due both to changes in the genome of a particular person during aging and to the lifetime determination of a particular repeat content in the genome that is unchanged throughout a person's life. In the second variant, we can propose a new prognostic marker of longevity.

Some assumptions about the change of the three analyzed genome repeats content during aging can be made in the study of replicative aging of cultured human cell lines. The data we obtained earlier for five lines of skin fibroblasts showed that during the aging process of each of the cell lines, the content of satellite III increases significantly and the content of telomere repeat decreases ([12]; (see also Figures 3(a) and 3(b)). A decrease in the telomere repeat content in natural and replicative aging has also been repeatedly described in the literature and is accepted as a generally recognized marker of aging and oxidative stress [60]. In two of the five cell lines, we recorded a decrease in the rDNA content at the latest passages [13]. With aging of cultured fibroblasts, only hypermethylated copies of rDNA are lost, if they were present in the genome of the young culture. These copies are not functional, contain numerous mutations. and complicate the process of rDNA replication in old cells.

The **S** and **T** parameters determined by the NQH method reflect only the average repeat content in the cell population and do not take into account the heterogeneity of cells in terms of this repeat content. The experiments with early passage fibroblast cloning have shown significant cellular heterogeneity in terms of satellite III content [14]. Different parts of the human brain DNA samples analysis demonstrated significant heterogeneity of CNS structures in terms of both satellite III and telomere repeat content against the background of stable rDNA content. The more satellite III contained a DNA sample, the fewer telomere repeats there were in this sample [74]. A negative correlation between the **S** and **T** parameters was also found in the present study (Figure 4).

Previously, we have shown that stem and differentiated cells with a high satellite III content have the characteristics typical for “old” cells: They do not divide and do not respond to various stimuli that induce an adaptive response in normal cells. Previously, the FISH method showed that an increase in the area of the hybridized satellite III in the cell is an early integral sign of cellular aging [46]. We further established that the observed increase in the signal area in this case occurs not only due to chromatin decondensation, as some authors believe, but also due to an increase of satellite III copy number itself [14].

The number of cells with a high satellite III content in a population depends on the intensity of the processes leading to the repeat accumulation in the cell and on the factors that eliminate such cells from the population. The mechanism of satellite III accumulation specifically during aging has not yet been described, but the mechanism of centromere satellite II augmentation in a cancer cell has been studied in detail. Centromere heterochromatin decondensation initiates this site transcription. DNA is synthesized on RNA using reverse transcriptase. RNA–DNA hybrids produced afterwards are embedded in chromatin, leading to activation of the repair process [79]. It is known that cellular aging is associated with significant transposon transcription activation [80]. Reverse transcriptase encoded by LINE produces RNA–DNA hybrids not only of LINE repeats but also of other genome sequences [81].

On the other hand, the cells with a large satellite III amount are not able to develop a sufficient adaptive response and therefore die due to a relatively low stress in the body with the following decrease of satellite content in DNA. In addition, some drugs can affect the accumulation and elimination of these cells from the population by blocking satellite transcription and autophagy activation [82]. A high level of oxidative stress may also block satellite III transcription and reduce satellite content in DNA [21]. Perhaps it can explain why some adults contain very low satellite III amounts in their DNA.

Unlike telomere and satellite III repeats, rDNA content in cells is a relatively stable genetic trait and is the same in all cells of the population and in various body tissues. According to our data, the **R** index does not depend on genotoxic factors and stress [21, 74] and can only occasionally change during carcinogenesis [83]. Apparently, parameter **R** does not change during human life, with the exception of possible rare and hitherto unproven cases (in 5%–10% of DNA samples) when the human genome contains hypermethylated rDNA copies, which can be eliminated not only during replicative aging but also, potentially, during natural aging. Based on this assumption, a significant narrowing of the rDNA range in the group of centenarians can possibly be explained by the fact that too high and too low rDNA copy numbers in the genome do not contribute to a long human life.

A low number of rDNA copies in the genome does not contribute to longevity, since it cannot provide a sufficient level of ribosome biogenesis for the body's response to stressful factors shortening life expectancy. Indeed, a low number of rDNA copies in the genome is associated with low stress resilience and an increased DNA damage. Previously, we found that elderly people over 60 years of age with a low number of rDNA copies in the genome are prone to the development of cognitive impairment and dementia [84] and this fact correlates with a decrease of ribosome biogenesis in such pathologies [85].

The negative impact produced on the lifespan by a large number of rDNA copies in the genome is not fully understood. We propose several hypotheses to explain this phenomenon. Firstly, protein synthesis, including ribosome biogenesis, is an energy-consuming process that requires large ATP reserves. Accordingly, other processes in the cell may be less efficient. Previously, a negative correlation was found between the number of rDNA copies in cells and the number of mitochondrial DNA copies [86].

Secondly, a large rDNA amount can accelerate aging, contributing to the accumulation of nonfunctional cells in the body with a large number of satellite III copies, which survive due to a high protein synthesis level. The accumulation of such cells in tissues disrupts the normal organism functioning. This assumption is supported by the data presented in Figures 4(b) and 4(c). An increase in the parameter **R** is associated with an increase in the line angle on the graph reflecting the dependence of **S** on **T**. If a cell population contains many rDNA copies, then a slight decrease in the average telomere length causes a significant increase in the satellite III content, which may indicate the relative stability of cells with a high content of satellite against the background of a large number of rDNA copies. Cells with a low rDNA content and a high satellite content die under the same conditions, and small changes in the satellite III content are recorded in the DNA.

Thirdly, the large rDNA amount in the genome may be due to the fact that the genome contains genetic features generally shortening the life expectancy and requiring an increased level of ribosome biogenesis for successful embryogenesis. Previously, we found an increased number of rDNA copies in the genomes of children with cystic fibrosis [87]. This monogenic disease caused by a mutation in the *CFTR* gene is associated with high oxidative stress levels. Possibly, high rDNA content in the genomes of such patients is associated with the low survival of embryos with *CFTR* gene mutation and low number of rDNA copies, which do not provide the necessary level of ribosome biogenesis during embryogenesis.

The maximum differences between the age groups are demonstrated by the calculated parameters **S**/**T** and **S**/(**R**∗**T**) (Figure 5(c)). Biologically, the **S** and **T** parameters are related. A decrease in telomere length causes decondensation of pericentromeric heterochromatin. Decondensation of heterochromatin leads to activation of satellite III transcription, which is one of the stages of the process leading to satellite III amplification. The **S/T** parameter reflects an increase in the leukocyte population, the number of cells with a high satellite content against the background of a decrease in the average telomere length. The **S/(R∗T)** parameter additionally takes into account a slight decrease in the rDNA content in older groups.

As the replicative aging experiments and analysis of primary human cells prove, these parameters are not stable genetic traits and constantly change during aging and under oxidative stress. Therefore, the **S**/**T** and **S**/(**R**∗**T**) parameters can potentially be used as prognostic markers of organism or cell line aging. The R parameter can be regarded as a potential genetic indicator of longevity, although further investigation is required to substantiate these hypotheses.

### 4.2. Strengths and Limitations of the Study

These data on the three tandem repeats' content in the DNA of people of different ages were obtained using the NQH method, developed in our laboratory specifically for highly repetitive tandem sequences analysis of various origins and qualities.

The NQH method can be attributed to direct methods of DNA detection. NQH does not employ Taq polymerase, so the results of the method are not significantly influenced by the DNA matrix quality. The initial DNA denatured with alkali hybridizes directly with a long labeled DNA fragment. The harsh hybridization and washing conditions allow determining only the desired fragment. Several control DNA samples with known tandem repeat content and nonhomologous DNA samples allow quantitative analysis and noise control. The result is not distorted by the degree of the DNA sample degradation [12].

In 2021 the authors of [11] for the first time applied the direct method (Oxford nanopore sequencing) of long DNA fragment sequencing without applying an amplification reaction for the analysis of rDNA in human DNA samples. According to this study, the number of rDNA copies in the sample of healthy people (*N* = 39) ranged from 250 to 700. This result is in good agreement with the data obtained by the NQH method in our present and previous studies [12, 13]. Ninety-six percent of the 535 DNA samples in the current study contain 250–700 rDNA copies (Figure 2(a)). The telomere repeat content determined in the present study also corresponds very well to the data obtained by other authors using direct analysis methods (pulsed-field gel electrophoresis). Therefore, the adequately used NQH method is allowing us to accurately determine the total highly repetitive tandem sequences content in DNA isolated from human cells.

The NQH method has several significant disadvantages. Firstly, it requires large amounts (100–200 ng of DNA per 1 assay) of well-purified DNA with a reliably measured concentration. Secondly, the method determines only the total average number of repeats in DNA isolated from a cell population, which may be heterogeneous in terms of the content of repeats in different cells, and is not able to estimate the polymorphic variants or pseudogene content.

## 5. Conclusion

The process of human aging is accompanied by alterations in the composition of three types of tandem repeats, namely ribosomal, satellite III (1q12), and telomere, within the DNA of peripheral blood leukocytes.

## Figures and Tables

**Table 1:** Demographic and clinical characteristics of the groups.

Group	*N*	Age	Gender: men	Never smoked-smoked-stopped smoking	Cancer	Cardiac ischemia	Cerebral circulation disorder
		±SD	%	%	*N*	*N*	*N*
I (5–17)	100	11.2 ± 2.5	62	96-3-1	—	—	—
II (18–44)	150	27.4 ± 6.7	56	89-7-4	—	—	—
III (45–74)	78	63.3 ± 10	47	59-24-17	—	—	—
IV (75–89)	101	81.2 ± 3.4	48	57-21-22	5	26	9
V (90–101)	106	92.7 ± 2.3	35	82-0-11	11	97	13

**Table 2:** Descriptive statistics reflecting the content of ribosomal, satellite, and telomere repeats (parameters **R**, **S,** and **T**, respectively**)** in the isolated DNA.

	Group	*N*	Mean	SD	Range	Median		CV
**R** Number of copies	I (5–17)	100	435	86	171–660	429	1	0.20
	II (18–44)	150	453	128	201–796	446	1.03	0.28
	III (45–74)	78	433	110	223–711	430	1.00	0.25
	IV (75–89)	101	401	71	202–603	393	0.92	0.18
	V (90–101)	106	405	56	289–535	404	0.94	0.14

**S** pg/ng DNA	I (5–17)	100	15	2	11–23	14	1	0.16
	II (18–44)	150	20	6	6–40	20	1.43	0.33
	III (45–74)	78	22	5	10–33	22	1.57	0.20
	IV (75–89)	101	20	7	6–34	21	1.51	0.34
	V (90–101)	106	25	7	12–44	25	1.79	0.27

**T** pg/μg DNA	I (5–17)	100	372	44	234–544	371	1	0.12
	II (18–44)	150	354	47	240–540	360	0.97	0.13
	III (45–74)	78	344	74	210–510	330	0.89	0.22
	IV (75–89)	101	268	90	120–570	250	0.67	0.34
	V (90–101)	106	249	55	94–374	253	0.68	0.23

**S/T** (∗100)	I (5–17)	100	4.0	0.8	2.2–8.4	3.9	1	0.20
	II (18–44)	150	5.7	1.9	1.5–10.5	5.6	1.44	0.33
	III (45–74)	78	6.7	1.9	3.1–13.8	6.6	1.69	0.29
	IV (75–89)	101	8.3	3.9	2.2–19.2	7.6	1.95	0.47
	V (90–101)	106	10.9	5.2	5.0–39.4	9.8	2.51	0.47

**S/(T∗R)** (∗10^5^)	I (5–17)	100	9.3	2.3	4.5–21.8	7.1	1	0.25
	II (18–44)	150	13.5	6.0	3.0–32.2	12.9	1.82	0.44
	III (45–74)	78	16.9	8.4	6.2–61.7	15.1	2.13	0.49
	IV (75–89)	101	20.8	9.0	4.3–44.2	19.7	2.77	0.43
	V (90–101)	106	27.5	12.9	12.6–86.9	24.9	3.51	0.47

Abbreviation: CV = coefficient of variation.

**Table 3:** Comparison of I–V groups by the variation of ribosomal (parameter *R*), satellite III (1q12) (parameter *S*), and telomere (parameter *T*) tandem repeats content.

Groups		Ribosomal repeat (R)				Satellite III (S)				Telomere repeat (Т)			
		Kolmogorov–Smirnov test		ROC analysis	*U*-test	Kolmogorov–Smirnov test		ROC analysis	*U*-test	Kolmogorov–Smirnov test		ROC analysis	*U*-test
X1	X2	D	α	AUC	*p*	D	α	AUC	*p*	D	α	AUC	*p*

I	II	−0.19	0.02	0.54	—	−0.59	10^–18^	0.78	< 10^−12^	0.27	0.0002	0.64	< 10^−3^
	III	0.18	0.11	0.51	—	−0.74	10^–21^	**0.90**	< 10^−20^	0.45	10^–8^	0.64	< 10^−3^
	IV	0.25	0.002	0.54	< 10^−3^	−0.52	10^–12^	0.74	< 10^−8^	0.71	10^–22^	**0.84**	< 10^−20^
	V	0.22	0.009	0.53	< 10^−2^	−0.76	10^–26^	**0.92**	< 10^−20^	0.84	10^–32^	**0.95**	< 10^−20^

II	III	0.10	0.29	0.55	—	−0.28	0.0005	0.62	< 10^−2^	0.30	0.0001	0.56	—
	IV	0.32	10^–6^	0.65	< 10^−3^	−0.12	0.32	0.52	—	0.57	10^–17^	**0.80**	< 10^−15^
	V	0.29	10^–5^	0.64	< 10^−3^	−0.32	10^–5^	0.70	< 10^−8^	0.72	10^–27^	**0.92**	< 10^−20^

III	IV	0.27	0.002	0.60	< 0.03	0.25	0.008	0.57	—	0.43	10^–7^	0.75	< 10^−8^
	V	0.30	0.0005	0.62	< 0.03	−0.31	0.0002	0.61	< 10^−2^	0.56	10^–12^	**0.84**	< 10^−15^

IV	V	−0.07	0.94	0.51	—	−0.25	0.002	0.67	< 10^−4^	0.19	0.048	0.52	—

**Groups**		**S/T**				**S/(T∗R)**							
		**Kolmogorov–Smirnov test**		**ROC analysis**	***U*-test**	**Kolmogorov–Smirnov test**		**ROC analysis**	***U*-test**				

X1	X2	D	α	AUC	*p*	D	α	AUC	*p*				

I	II	−0.63	10^–21^	0.79	< 10^−14^	−0.54	10^–15^	0.71	< 10^−8^				
	III	−0.79	10^–24^	**0.92**	< 10^−20^	−0.72	10^–20^	**0.88**	< 10^−20^				
	IV	−0.73	10^–23^	**0.88**	< 10^−20^	−0.82	10^–30^	**0.92**	< 10^−20^				
	V	−0.94	10^–40^	**0.98**	< 10^−20^	−0.96	10^–42^	**0.99**	< 10^−20^				

II	III	−0.26	0.001	0.64	< 10^−3^	−0.22	0.012	0.63	< 10^−2^				
	IV	−0.36	10^–6^	0.70	< 10^−7^	−0.40	10^–8^	0.75	< 10^−10^				
	V	−0.60	10^–10^	**0.89**	< 10^−20^	−0.60	10^–19^	**0.87**	< 10^−20^				

III	IV	−0.31	0.0003	0.60	—	−0.27	0.003	0.64	< 10^−2^				
	V	−0.56	10^–12^	**0.82**	< 10^−13^	−0.44	10^–7^	0.78	< 10^−11^				

IV	V	−0.27	0.0003	0.66	< 10^−4^	−0.25	0.002	0.66	< 10^−4^				

*Note:* Bold values indicate *p* < 0.001.

## Data Availability

The data that support the findings of this study are available from the corresponding author upon reasonable request.

## Supporting Information

Supporting data illustrate the dependence of ribosomal (parameter **R**), satellite III (1q12) (parameter **S**), and telomere (parameter **T**) tandem repeats content on age. Trend line equations and Spearman statistics data are included in the illustration.
