# Supplementary figures and images for: Variation in the Content of Three Tandem Repeats of the Human Genome (Ribosomal, Satellite III, and Telomere) in Peripheral Blood Leukocyte DNA of People of Different Ages (5–101 Years)
Source: J Aging Res. 2025 Sep 11;2025:8847073. doi: 10.1155/jare/8847073 (PMC12446595; doi:10.1155/jare/8847073)

**R**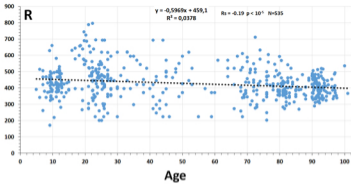**T**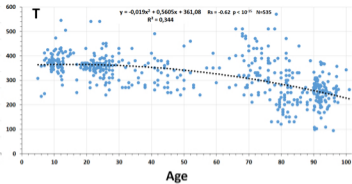**S**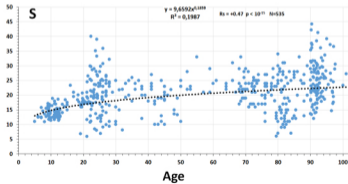**S**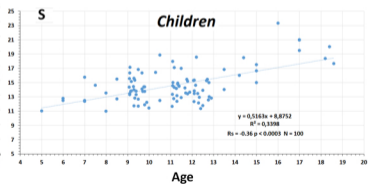

Supplement: Supporting Information — Additional supporting information can be found online in the Supporting Information section. [file 8847073.f1.pdf]
